# Hydrogen Sulfide Delivery in Sulfurous Thermal Waters: Saturnia as a Translational Model for Gasotransmitter Pharmacology—A Narrative Review

**DOI:** 10.1002/hsr2.72919

**Published:** 2026-08-04

**Authors:** Elisabetta Ferrara, Manela Scaramuzzino, Davide Ciaramellano, Luigi Brunetti, Giuseppe Balice, Giovanna Murmura, Bruna Sinjari

**Affiliations:** ^1^ Telematic University Leonardo Da Vinci, UNIDAV Torrevecchia Teatina Italy; ^2^ Medical Thermal Center of Saturnia Grosseto Italy; ^3^ Department of Pharmacology G. D'Annunzio University of Chieti‐Pescara Chieti‐Pescara Italy; ^4^ Unit of Prosthodontics, Department of Innovative Technologies in Medicine and Dentistry University “G. d'Annunzio” of Chieti‐Pescara Chieti Italy

**Keywords:** balneotherapy, bioglea, gasotransmitter, hydrogen sulfide, microbial interface, saturnia, sulfurous thermal waters, translational pharmacology

## Abstract

**Background:**

Hydrogen sulfide (H2S) is an endogenous gasotransmitter regulating redox balance, inflammation, and vascular tone, but reproducing the spatial and kinetic features of endogenous production remains challenging. We examine sulfurous thermal waters as natural contexts of sustained H2S exposure, using the Saturnia springs (Tuscany, Italy) as a representative natural analog to discuss geochemical determinants of sulfide stability, the bioglea microbial interface, and the clinical evidence base.

**Methods:**

We conducted a structured, non‐systematic literature search of PubMed, Scopus, and Web of Science (inception to March 2026) using predefined sulfide‐, balneology‐, and Saturnia‐related terms. Experimental, observational, geochemical, and review sources were narratively synthesized and critically appraised; no meta‐analysis or formal quality scoring was performed, consistent with the hypothesis‐generating scope.

**Results:**

Saturnia waters show elevated, relatively stable sulfide (≈14 mg/L; historical range ≈13–20 mg/L) within a buffered carbonate matrix. At pH ~6.85, a transparent Henderson–Hasselbalch calculation indicates that roughly 45%–55% of total dissolved sulfide is present as undissociated molecular H_2_S, depending on temperature and ionic strength. Controlled hydropinotherapy studies and sulfurous insufflation protocols suggest measurable effects on surrogate outcomes related to oxidative stress and to auditory and respiratory function, but the literature is limited by small sample sizes, heterogeneous exposure modalities, and a scarcity of trials with standardized clinical endpoints.

**Conclusions:**

Rather than a validated translational model, Saturnia offers an informative, hypothesis‐generating natural analog for sustained H2S exposure in complex aqueous matrices, from which generalizable design principles and translational priorities—including route‐specific pharmacokinetics and standardized exposure reporting—can be abstracted for gasotransmitter delivery.

## 1 Introduction

Hydrogen sulfide (H_2_S) has undergone a profound paradigm shift in biomedical science, transitioning from its former classification as a toxic environmental pollutant to recognition as a crucial endogenous gasotransmitter alongside nitric oxide and carbon monoxide [1]. This reevaluation has catalyzed extensive research into the role of H_2_S in physiological signaling and therapeutic applications [2]. However, significant challenges remain in developing controlled delivery systems capable of reproducing the spatial specificity and bioavailability of endogenous gasotransmitter production [3]. Natural geothermal systems represent unique physicochemical models in which geological, geochemical, and microbiological processes converge, resulting in sustained gasotransmitter delivery mechanisms shaped over geological timescales [4]. Among these systems, the Saturnia thermal springs (Tuscany, Italy) provide a well‐characterized case study for examining sustained H_2_S exposure in a natural thermal environment, with continuous documented utilization spanning more than two millennia [5]. Comprehensive geochemical and isotopic investigations have characterized this thermal system in detail, revealing waters that emerge at 34.1°C–37.5°C with reported total dissolved sulfide concentrations of approximately 14 mg/L (expressed as H_2_S equivalents), with higher values described under specific analytical and sampling conditions [6]. Throughout this review, “total sulfide” (ΣS^2−^) denotes the sum of dissolved sulfide species (H_2_S + HS^−^ + S^2−^), whereas “H_2_S” refers specifically to the undissociated molecular form, which predominates alongside HS^−^ at the near‐neutral pH of these waters; the negligible S^2−^ fraction is omitted from speciation estimates given its very high pKa_2_ [6]. The broader terms “sulfurous” or “sulfur‐containing compounds” are used only where the wider chemical category (including sulfate and organic sulfur species) is intended, and are distinguished from dissolved sulfide species throughout. These concentrations are higher than those typically reported for sulfurous therapeutic springs and fall within ranges discussed as potentially relevant in balneological studies [7]. The Saturnia waters are highly mineralized and characterized by elevated dissolved sulfide concentrations and stable physicochemical conditions [6]. Hydrogeological investigations further support a deep circulation system conducive to sulfide stability and persistence [6]. The δ^13^C value of −1.54‰ suggests limited atmospheric infiltration, creating physicochemical conditions conducive to sulfide stability and consistent delivery. The therapeutic use of sulfurous thermal waters predates contemporary biochemistry by millennia; however, the molecular mechanisms underlying their reported efficacy have only recently become the subject of investigation [8, 9]. In contrast to synthetic H_2_S donors commonly employed in laboratory research, which often exhibit rapid bolus release or limited control over release kinetics, the Saturnia system provides H_2_S within a complex physicochemical matrix that may influence sulfide speciation, stability, and potential bioavailability [10]. The therapeutic profile associated with H_2_S exposure encompasses multiple physiological targets through concentration dependent mechanisms. At physiologically relevant concentrations, H_2_S acts as an antioxidant, anti‐inflammatory agent, and vasodilator, while regulating cellular processes including proliferation, apoptosis, and differentiation [9]. This mechanistic versatility underpins reported therapeutic applications across dermatological, rheumatological, and respiratory conditions [11]. A distinguishing feature of the Saturnia system is the development of a complex microbial ecosystem—termed bioglea—at the water–atmosphere interface [12]. This stratified microbial community, composed primarily of cyanobacteria alongside sulfate‐reducing bacteria and anoxygenic phototrophs, constitutes a dynamic biological interface that modulates H_2_S bioavailability while synthesizing complementary bioactive compounds [12, 13]. The presence of this living component introduces an additional biological interface that may influence local sulfide dynamics and associated bioactivity. This narrative review critically examines current evidence on H_2_S exposure in natural sulfurous thermal environments, using Saturnia as a representative natural analog—rather than a validated translational model—to discuss geochemical determinants, microbial interfaces, and key gaps relevant to translational gasotransmitter research.

## 2 Literature Search and Study Selection

This narrative review was conducted following the principles of the Scale for the Assessment of Narrative Review Articles (SANRA) to ensure methodological transparency within the limits of a non‐systematic design. A structured, non‐systematic literature search was performed in three electronic databases—PubMed/MEDLINE, Scopus, and Web of Science—covering the period from each database's inception to March 2026, with the search updated during manuscript revision to incorporate recently published Saturnia‐specific studies. The search combined controlled vocabulary and free‐text terms across three conceptual domains (sulfur chemistry/biology; balneological and thermal context; and mechanistic or clinical outcomes). A representative full search string used in PubMed was: *(“hydrogen sulfide”[MeSH] OR “hydrogen sulfide” OR “H_2_S” OR “dissolved sulfide” OR “total sulfide”) AND (“thermal water*” OR “balneotherapy” OR “balneology” OR “sulfurous spring” OR “Saturnia” OR “mineral water”) AND (“gasotransmitter*“ OR “oxidative stress” OR “anti‐inflammatory” OR “microbial mat” OR “biofilm” OR “bioglea” OR “clinical outcome”). Equivalent strings, adapted to the syntax of each database, were applied in Scopus and Web of Science. Reference lists of retrieved articles and relevant reviews were screened manually to identify additional sources not captured by the database search. Eligibility was guided by predefined inclusion and exclusion criteria. Sources were included if they were peer‐reviewed and addressed at least one of the review's conceptual domains—sulfurous‐water composition and geochemistry, microbial ecology of thermal systems, H_2_S‐related molecular mechanisms, or reported clinical/therapeutic outcomes—and comprised experimental studies, observational clinical investigations, geochemical and environmental analyses, or authoritative reviews. English‐language articles were prioritized; earlier seminal or Saturnia‐specific sources in other languages were retained when relevant to the historical or geochemical context, given the limited and largely Italian primary literature on this specific system. Sources were excluded if they were case reports, conference abstracts, editorials, or non‐peer‐reviewed or gray literature. Because Saturnia‐specific primary studies are scarce, broader sulfurous‐balneology and H_2_S‐biology sources were included to contextualize findings, with this trade‐off between specificity and coverage acknowledged as a limitation of the narrative approach. Evidence was synthesized narratively and appraised critically, with attention to study design, exposure characteristics, endpoint type (validated clinical endpoint vs*.* surrogate biomarker), and acknowledged limitations. Given the hypothesis‐generating scope of the review, no formal screening counts, PRISMA flow diagram, meta‐analysis, or quantitative quality scoring were performed; this is acknowledged as a methodological limitation rather than presented as a systematic synthesis.

### 2.1 Geological and Geochemical Framework of the Saturnia System

The Saturnia thermal system originates within the southern Tuscany extensional province, where post‐collisional tectonics have created favorable conditions for deep groundwater circulation and prolonged geochemical evolution [14]. The system is hosted within a fractured carbonate aquifer associated with the Calcare Cavernoso formation and has been described as exhibiting high physicochemical stability in geochemical and hydrogeological investigations [6]. Thermal waters emerge at a nearly constant temperature of approximately 34°C–37.5°C, suggesting limited conductive heat loss during ascent through preferential flow paths within the carbonate aquifer [6]. Such thermal constancy may contribute to the persistence of dissolved hydrogen sulfide (H_2_S) and to the establishment of microbial communities involved in sulfur metabolism at the water–atmosphere interface [12].

Electrical conductivity values around 2890 μS/cm indicate substantial mineralization resulting from extended water–rock interactions during deep circulation [6]. Reported total dissolved sulfide concentrations in Saturnia have been described in the range of approximately 13–20 mg/L across historical analytical campaigns, with typical recent values around 14–15 mg/L depending on sampling conditions and degassing [6]. These values place Saturnia among the most sulfur‐rich therapeutic springs reported in Europe and approach the upper range of concentrations proposed in balneological studies [6, 7]. A methodological caveat applies to the sulfide concentrations reported here. Peer‐reviewed hydrogeochemical characterizations [6] and the operator's official analytical profile are not fully equivalent sources: the former provide isotopic and multi‐element data under documented analytical conditions, whereas the latter reflects routine monitoring and is reported here only for parameters where peer‐reviewed values are unavailable or to illustrate the typical operational profile. Dissolved sulfide is, moreover, intrinsically difficult to quantify reproducibly: H_2_S is volatile and readily lost through degassing during sampling, transport, and analysis, and measured values are sensitive to sampling depth, temperature, oxygenation, and the time elapsed before fixation. Reported concentrations should therefore be regarded as operationally defined estimates rather than exact values, and the historical range (≈13–20 mg/L) plausibly reflects genuine temporal variation as well as differences in sampling and analytical protocols. This measurement uncertainty propagates to any estimate of bioaccessible mole. The water pH of approximately 6.85 is relevant for sulfide speciation and potential bioavailability. Applying the Henderson–Hasselbalch relationship with a temperature‐corrected first dissociation constant (pKa_1_ ≈ 6.98 at 25°C and ≈ 6.77 at 37°C), roughly 45%–55% of total dissolved sulfide is estimated to be present as undissociated molecular H_2_S rather than the hydrosulfide anion (HS^−^) at this pH. This fraction is approximate and depends on temperature and matrix ionic strength; it is presented as a transparent calculation rather than a direct measurement. The substantial molecular fraction may favor percutaneous and inhalational uptake during exposure [7]. These concentrations exceed those commonly reported for sulfurous thermal waters (generally 1–5 mg/L), highlighting the distinctive sulfide content of the Saturnia system.

Isotopic and geochemical studies further suggest long groundwater residence times and limited interaction with recent meteoric inputs, conditions that may favor sustained sulfide stability and chemical buffering [6]. Strontium isotopic signatures link the circulating waters to the Calcare Cavernoso limestone, while carbon and oxygen isotope data indicate extensive interaction with carbonate minerals and recharge from elevated carbonate outcrops south of Roccalbegna. Together, these features are consistent with a model of deep, geochemically isolated circulation conducive to reducing conditions and sulfide preservation [6].

H_2_S generation within the Saturnia system has been attributed to multiple interconnected pathways operating at different depths. Thermochemical sulfate reduction associated with evaporitic formations has been proposed as a primary abiotic sulfide source, while additional biotic contributions have been hypothesized under conditions permitting microbial activity [15]. These processes establish a continuum between geochemical and biological sulfur cycling, providing the basis for the development of the complex bioglea ecosystem at the surface.

Beyond sulfide, the waters display a characteristic mineral matrix, including elevated concentrations of sulfate, bicarbonate, calcium, strontium, boron, lithium, and trace elements. Selenium concentrations reported for the springs and associated wells exceed typical groundwater values, suggesting contributions from deep geological sources during circulation. Rather than acting independently, these chemical components collectively influence redox stability, buffering capacity, and sulfide persistence [6, 7].

Long‐term monitoring data reported by regional authorities indicate limited compositional variability over multiple decades, supporting the interpretation of Saturnia as a relatively stable natural system for therapeutic H_2_S exposure [6]. Selected historical monitoring data from the Regione Toscana environmental database are summarized in Table 1.

**Table 1:** Representative physicochemical composition of Saturnia thermal waters (official monitoring profile).

Parameter	Value
Temperature	37.5°C
pH (18°C)	6.85 ± 0.05
Conductivity	2850 ± 50 µS/cm
Fixed residue (180°C)	2790 mg/L
Total sulfide	(ΣS^2−^, as H_2_S) | 14.0 ± 1.2 mg/L
SO_4_^2−^	1475 ± 5 mg/L
HCO_3_^−^	637 mg/L
Ca^2+^	561 ± 39 mg/L
Mg^2+^	130 mg/L
Na^+^	71 mg/L
B	19.3 mg/L
Li^+^	0.6 mg/L
Sr^2+^	7.3 mg/L
CO_2_ (total)	728 mg/L

*Note:* Representative physicochemical parameters and major ion composition of the Saturnia thermal spring waters. Values are compiled from the official analytical profile provided by Terme di Saturnia (Tuscany, Italy) and, where available, cross‐referenced with peer‐reviewed hydrogeochemical data [6]; isotopic and trace‐element values derive from the latter. Values are expressed at the sampling conditions indicated (e.g., pH measured at 18°C) and describe the typical mineralization and dissolved sulfide content of the system. Total sulfide is reported as the analytically determined sum of dissolved sulfide species (H_2_S + HS^−^), expressed as H_2_S equivalents; the value therefore reflects total dissolved sulfide rather than the molecular H_2_S fraction alone. At the measured pH of 6.85, the undissociated molecular H_2_S fraction is estimated at approximately 45%–55% of total dissolved sulfide, based on the Henderson–Hasselbalch relationship with a temperature‐corrected pKa_1_ (see text); this is an approximate estimate, dependent on temperature and matrix ionic strength, and not a direct measurement.

Overall, the integrated geochemical profile of the Saturnia system suggests that reported biological effects may not depend solely on H_2_S concentration, but also on the broader physicochemical matrix, including temperature, pH, ionic composition, trace elements, and dissolved gases. This naturally buffered environment may provide sustained H_2_S delivery within a relatively narrow concentration range and differs from synthetic H_2_S donors by offering intrinsic stabilization mechanisms and potentially synergistic chemical conditions that could influence biological uptake and activity [8].

### 2.2 The Bioglea Interface as a Biological Modulator of Sulfide Availability

#### 2.2.1 Empirically Supported Findings

The elevated and relatively stable dissolved sulfide concentrations reported for Saturnia waters create a distinctive ecological niche that supports the development of specialized microbial communities at the water–atmosphere interface [6]. These conditions give rise to the bioglea, a stratified, cyanobacteria‐dominated microbial mat—primarily of the order Oscillatoriales (e.g., Oscillatoria sp., Spirulina cf. labyrinthiformis)—that forms a multilayered biofilm over the pool sediment and detaches to float at the surface during the day, as described in Saturnia‐specific biological investigations [12, 13]. Chromatographic and spectrometric analyses (HPLC, GC‐MS, SPME) have identified diverse classes of organic compounds in bioglea extracts, summarized in Table 2, including fatty acids, sterols, amino acids, phenolic compounds, hydrocarbons, and photosynthetic pigments such as chlorophyll *a*, carotenoids, and phycobiliproteins [12, 13]. Bioglea extracts have demonstrated measurable antioxidant activity across three independent in vitro assays (DPPH, ORAC, ABTS), with hydrophilic extracts showing the highest radical‐scavenging capacity; this activity has been attributed primarily to phycocyanin, amino acids, and polyphenols [12]. These empirically established findings position the bioglea as a source of bioactive compounds with a characterized chemical composition and demonstrated in vitro antioxidant activity.

**Table 2:** Classes of bioactive compounds reported in the Saturnia bioglea.

Compound class	Representative components identified	Analytical method
Fatty acids	Saturated (C9–C20; palmitic predominant) and unsaturated (oleic) fatty acids; odd‐chain (C15, C17)	GC‐MS
Hydroxy and dicarboxylic acids	Hydroxy acids, dicarboxylic acids	GC‐MS
Sterols	Cholesterol, β‐sitosterol, stigmasterol, desmosterol	GC‐MS
Pigments	Chlorophyll a; carotenoids; phycocyanin; phycoerythrin	HPLC/UV‐Vis
Amino acids	Essential l‐amino acids (e.g., L‐isoleucine, l‐phenylalanine, L‐leucine)	GC‐MS
Phenolic/aromatic compounds	Phenols, benzoic/cinnamic acid derivatives	GC‐MS
Hydrocarbons and alcohols (volatiles)	Hydrocarbons (C11–C17); long‐chain alcohols (C12–C16); limonene, p‐cresol	SPME‐GC‐MS
Terpenoids	Squalene, phytol, lycopersene	GC‐MS

*Note:* Compounds identified in bioglea samples from the Saturnia thermal pool by Centini et al. [12]. Detection is qualitative; relative proportions were estimated from chromatographic peak areas. Antioxidant (radical‐scavenging) activity of bioglea extracts was confirmed by DPPH, ORAC, and ABTS assays, with hydrophilic extracts most active [12]. Pigment composition (chlorophyll a, carotenoids, phycobiliproteins) was further characterized in [13].

### 2.3 Hypotheses and Proposed Mechanisms

Beyond these documented findings, several mechanistic roles for the bioglea in modulating sulfide dynamics remain hypothetical and are presented here as explicitly labeled conjectures requiring empirical validation. A documented starting point is that several bioglea cyanobacteria—notably *Spirulina cf. labyrinthiformis*—are capable of anoxygenic photosynthesis using H_2_S as an electron donor, excreting elemental sulfur granules visible within the filaments [12]. This establishes a direct, demonstrated link between the microbial community and local sulfur cycling. Building on this, it is plausible—but not yet quantified in situ—that the bioglea actively shapes the dissolved sulfide budget at the water–atmosphere interface: sulfide consumption by anoxygenic phototrophs during daylight, set against sulfide regeneration by sulfate‐reducing bacteria under the reducing conditions typical of such mats [16], could generate diurnal and spatial gradients in sulfide concentration and speciation precisely at the depth where bathers are exposed. Second, the bioglea may generate a localized redox and chemical microenvironment at the skin–water boundary that further modulates sulfide bioavailability during immersion; this proposed interface effect has not been directly measured. Third, because the Saturnia pools are open bathing environments, exogenous and endogenous bioactive substances may be reciprocally adsorbed within the microbial biofilm [13], potentially altering the bioactive milieu experienced by the skin.

These hypotheses generate testable predictions. (i) If anoxygenic phototrophs and sulfate‐reducing bacteria jointly govern interface sulfide levels, then dissolved sulfide concentrations measured immediately above an intact bioglea mat should show measurable diurnal variation (light‐dependent depletion) and differ from bioglea‐free water under matched temperature and depth conditions; in situ sulfide microelectrode profiling across a light–dark cycle would test this directly. (ii) If the bioglea exerts an antioxidant or anti‐inflammatory interface effect, then ex vivo skin or cellular models exposed to sulfurous water in the presence versus absence of bioglea extract should show differential redox or inflammatory marker responses. Targeted quantitative metagenomic profiling, paired with microelectrode measurements, would be required to test these predictions, neither of which is currently available for the Saturnia bioglea [12, 13].

### 2.4 Molecular Basis of H_2_S Bioactivity and Delivery

At physiologically relevant concentrations, H_2_S modulates cellular signaling primarily through protein persulfidation across multiple biological systems. These pathways are relevant when considering exogenous exposure scenarios, including sulfurous thermal waters, in which H_2_S concentration, physicochemical context, and exposure dynamics may influence biological activity and therapeutic potential.

Due to its small size and physicochemical properties, H_2_S can diffuse across biological membranes and exert intracellular effects. At physiologically relevant concentrations, its actions are largely mediated through protein sulfhydration (persulfidation), a reversible post‐translational modification of reactive cysteine residues that modulates the function of proteins involved in cellular metabolism, antioxidant defense, and signal transduction [17, 18].

Additional molecular targets reported in the literature include ATP‐sensitive potassium channels, whose activation has been associated with vasodilation, as well as redox‐sensitive enzymes and transcriptional regulators implicated in antioxidant and cytoprotective responses [19, 20]. At higher concentrations, H_2_S has been shown to reversibly inhibit mitochondrial respiration through interaction with cytochrome c oxidase, resulting in a concentration‐dependent transition from physiological signaling to metabolic inhibition [21]. This biphasic behavior is often described as a hormetic response, characterized by a relatively narrow window in which antioxidant, anti‐inflammatory, and vasodilatory effects predominate, beyond which cellular toxicity may emerge [19]. The biological effects of exogenous H_2_S are strongly influenced by delivery characteristics and environmental context. Compared with fast‐releasing synthetic donors, which can generate transient peaks in tissue H_2_S levels, sustained or buffered exposure has been proposed to favor signaling pathways associated with longer‐lasting antioxidant and anti‐inflammatory effects [22]. Environmental factors such as temperature, pH, redox conditions, and mineral composition further modulate H_2_S stability, speciation, and bioavailability, particularly in complex aqueous matrices. In the Saturnia system, the combination of elevated temperature (~37.5°C) and mildly acidic pH (~6.85) favors the persistence of an appreciable molecular H_2_S fraction and may enhance percutaneous and inhalational exposure, potentially contributing to sustained concentrations within ranges discussed in the balneological literature [6, 7, 9]. Although direct pharmacokinetic measurements during balneotherapeutic exposure remain limited, experimental and translational evidence supports the plausibility that such exposure may allow biologically relevant signaling under typical conditions [23].

### 2.5 Clinical Applications, Safety, and Research Priorities

Clinical evidence regarding sulfurous thermal therapy at Saturnia spans multiple medical domains, but remains limited overall by small sample sizes, heterogeneous exposure protocols, and a scarcity of randomized trials with standardized clinical endpoints. The available evidence can be grouped into three tiers of methodological strength. The strongest comprises small controlled studies of oral hydropinotherapy in healthy volunteers (Pagliarani et al.; Benedetti et al.), which report antioxidant effects but rely on surrogate biochemical markers rather than clinical endpoints. An intermediate tier comprises uncontrolled observational studies with validated clinical or patient‐reported endpoints but no comparator group (Nappi et al.; Ferrara et al., 2025, 2026a, 2026b). The weakest comprises hypothesis‐generating observations, including data collected opportunistically during service interruptions. Across all three tiers, the same limitations recur—small and often non‐representative samples, heterogeneous exposure modalities and outcome measures, absence of randomization or active comparators, and reliance on surrogate or self‐reported endpoints—and these constraints are emphasized consistently in the appraisal that follows. In appraising this evidence, we distinguish throughout between studies reporting validated clinical endpoints (e.g. audiometric thresholds, validated patient‐reported outcome measures) and those relying on surrogate biomarkers (e.g. circulating oxidative‐stress markers), noting for each whether a comparator group was present; several recent studies employ validated patient‐reported outcome measures within uncontrolled designs, in which instrument validity does not compensate for the absence of a comparator. This distinction is summarized in Table 3. In otorhinolaryngological settings, Nappi et al. evaluated 15 adult patients with rhinogenic hearing loss treated with a cycle of 12 sulfurous endotympanic insufflations and reported significant improvements in audiometric thresholds across the 250–2000 Hz range—the frequencies most relevant to communication — whereas changes at higher frequencies (4000–8000 Hz) did not reach significance. While these findings suggest potential benefit, interpretation is limited by the small, non‐randomized, uncontrolled single‐center design and the absence of a comparator group [24]. While these findings suggest potential clinical benefit, interpretation would be strengthened by clearer reporting of effect sizes, standardized outcome definitions, and systematic adverse event monitoring. Dermatological applications of sulfurous immersion and mud therapy have been associated with anti‐inflammatory effects, improvements in microcirculation, and enhancement of skin barrier function, consistent with the known biological actions of dissolved sulfide and sulfate [25]. However, most controlled evidence derives from studies conducted at various European sulfurous spas, and robust Saturnia‐specific investigations for dermatological indications remain limited. Across the broader literature, heterogeneity in disease severity, concomitant interventions, and exposure modalities (immersion, mud packs, inhalations) complicates direct comparison and prevents firm conclusions regarding indication‐specific efficacy. Evidence regarding therapeutic durability and treatment continuity remains scarce. Observational data collected during COVID‐19–related service interruptions at Saturnia reported clinical worsening in a proportion of patients following discontinuation of thermal therapy, with recovery trajectories influenced by prior exposure duration [26]. Although these findings are subject to confounding factors inherent to single‐center observational designs, they raise the hypothesis that repeated exposure may be associated with sustained effects and underscore the need for prospective longitudinal studies. More recently, two retrospective real‐world analyses conducted at Saturnia have extended the range of outcomes examined to patient‐reported quality of life and sleep. In a single‐arm pre–post study of 76 participants undergoing a 7–12‐day immersion cycle, self‐reported insomnia severity and sleep satisfaction improved significantly among individuals with baseline sleep disturbance, although the authors explicitly attributed these changes to the multi‐component spa context rather than to sulfide‐specific effects, given the absence of a heated non‐mineral water comparator [27]. A larger retrospective analysis of 190 patients similarly reported significant improvements in SF‐36 and WHOQOL‐BREF domains, with superior outcomes under sustained versus acute exposure; a structured exploratory framework identified H_2_S as the component with the strongest mechanistic plausibility, while emphasizing that the single‐source design precluded any causal or component‐specific attribution [28]. Both studies share the methodological constraints intrinsic to observational, single‐center designs without control groups and should be read as hypothesis‐generating; notably, several of the present authors were involved in these investigations, and this potential interpretive bias is acknowledged. Water temperature near physiological levels (approximately 37.5°C at Saturnia) may enhance local circulation and membrane permeability, potentially influencing exposure dynamics, while the chemical component plausibly contributes through sulfide‐mediated signaling pathways, including antioxidant and anti‐inflammatory responses. Direct comparative studies designed to disentangle thermal versus chemical contributions, however, remain scarce. Across the available studies, adverse event reporting is inconsistent and rarely systematic. Most reports describe sulfurous thermal therapy at Saturnia as well tolerated, with only minor and transient effects (e.g. localized skin irritation) noted in some cohorts [29, 30], but few studies applied a predefined adverse event taxonomy, structured solicitation, or standardized severity grading, and none reported active long‐term safety surveillance. This absence of harmonized safety monitoring represents a methodological gap, particularly relevant for vulnerable populations (e.g. patients with cardiovascular or respiratory comorbidity), in whom both sulfide exposure and thermal load warrant explicit assessment. Future protocols should incorporate prospective, standardized adverse event capture alongside efficacy endpoints. A recurring limitation across the clinical literature is the variability of treatment modalities and exposure parameters, including route of administration (immersion, hydropinotherapy, inhalations, mud therapy), session duration, cycle structure, and total treatment period. This heterogeneity limits evidence synthesis and underscores the importance of standardized protocol reporting. Future investigations should specify water temperature at the point of exposure, measured or estimated dissolved sulfide content concentrations (accounting for degassing and sampling variability), exposure frequency and duration, total treatment cycle length, and concomitant interventions. Harmonized clinical endpoints—including validated patient‐reported outcome measures, objective functional assessments, and standardized biomarker panels—would facilitate cross‐study comparison and enable more robust meta‐analytic approaches. The development of a core outcome set for sulfurous balneotherapy research represents a priority for the field. The main controlled and observational clinical studies available to date are summarized in Table 3.

**Table 3:** Clinical studies on saturnia sulfurous thermal waters and reported outcomes.

Study (Author, Year)	Study design	Population	Exposure modality	Main outcomes reported	Endpoint type	Key limitations
Nappi et al., 1995	Uncontrolled observational	15 adults with rhinogenic hearing loss (mean age 29 y; 9 transmissive, 6 mixed)	Sulfurous endotympanic insufflation (12 sessions)	Significant audiometric threshold improvement at 250–2000 Hz; non‐significant at 4000–8000 Hz	Validated clinical endpoint (audiometry)	Small sample; non‐randomized; uncontrolled; single‐center
Pagliarani et al., 2005	Controlled	21 healthy volunteers	Oral hydropinotherapy (500 mL/day, 15 days)	↓ lipid peroxidation markers; ↑ antioxidant potential up to 15 days post‐treatment	Surrogate biomarker	Healthy population; surrogate endpoints; no pharmacokinetic assessment
Benedetti et al., 2009	Controlled (study group vs controls)	40 healthy adults	Oral hydropinotherapy (500 mL/day, 2 weeks)	↓ malondialdehyde, carbonyls, AOPP; ↑ total antioxidant capacity; ↑ plasma thiols	Surrogate biomarker	Healthy population; short follow‐up; randomisation not specified; no systemic uptake measurements
Ferrara et al., 2025	Observational (single‐center)	Patients receiving long‐term thermal therapy	Balneotherapy + mud + inhalations (12–15 sessions/cycle)	Symptom worsening after discontinuation during COVID‐19 interruption; recovery influenced by prior exposure duration	Validated PROMs; hypothesis‐generating	Single‐center; strong confounding; no standardized endpoints
Ferrara et al., 2026a	Single‐arm observational (pre–post)	76 spa visitors	Sulfurous immersion (60–90 min/session, 7–12 days)	↓ OSQ insomnia severity and ↑ sleep satisfaction in participants with baseline disturbance; no change in non‐disturbed subgroup (ceiling effect)	Validated PROM (OSQ); uncontrolled	Single‐arm; no control group; self‐report only; non‐specific spa/vacation confounders
Ferrara et al., 2026b	Retrospective observational (stratified)	190 patients	Sulfurous immersion (acute 2–3 h vs. sustained 3–14 days)	↑ SF‐36 PCS and WHOQOL‐BREF physical domain; superior outcomes with sustained exposure; H_2_S identified as priority candidate component	Validated PROMs (SF‐36, WHOQOL‐BREF); uncontrolled	Single‐source design; no causal/component‐specific attribution; retrospective

*Note:* Studies are ordered chronologically. Endpoint type distinguishes validated clinical or patient‐reported endpoints from surrogate biochemical markers; design and comparator status are indicated to reflect the methodological hierarchy discussed in the text.

Abbreviations: OSQ, Oviedo Sleep Questionnaire; PROM, patient‐reported outcome measure; SF‐36 PCS, Short Form‐36 Physical Component Summary; WHOQOL‐BREF, World Health Organization Quality of Life–BREF.

## 3 Conclusions and Future Directions

Hydrogen sulfide (H_2_S) is increasingly recognized as an endogenous mediator involved in redox regulation, inflammatory signaling, and vascular homeostasis. Natural sulfurous thermal environments provide an uncommon exposure context in which dissolved sulfide is delivered in a complex physicochemical and biological matrix. The Saturnia thermal springs represent a well‐characterized example, combining elevated and relatively stable sulfide concentrations with long‐term geochemical buffering and the presence of a biologically active microbial interface (bioglea) that may influence local sulfur dynamics.

Available experimental and clinical observations suggest that sulfurous water exposure may be associated with measurable modulation of oxidative stress–related pathways and selected respiratory outcomes. However, the current evidence base remains limited by small study populations, heterogeneous treatment modalities, frequent reliance on surrogate biomarkers, and the scarcity of rigorously designed randomized trials with standardized clinical endpoints. Quantitative pharmacokinetic data defining systemic H_2_S uptake across immersion, inhalation, and oral hydropinotherapy are also largely lacking.

Rather than a validated translational model—a claim that would require systemic pharmacokinetic and exposure‐modeling data not yet available—Saturnia is better understood as a natural analog from which generalizable principles for gasotransmitter delivery can be abstracted. Four such principles emerge. The first is matrix buffering: a carbonate‐buffered, mineral‐rich matrix maintains dissolved sulfide within a narrow, relatively stable concentration window, in contrast to the bolus kinetics of most synthetic donors. The second is sustained exposure: continuous low‐level delivery over repeated sessions, rather than transient peaks, may favor signaling‐range rather than inhibitory‐range effects. The third is interface modulation: a living biological interface (the bioglea) may locally shape sulfide speciation and bioavailability at the point of contact, suggesting that engineered delivery systems could incorporate analogous interfacial control. The fourth concerns route‐specific constraints: percutaneous and inhalational uptake are governed by the molecular H_2_S fraction, which is in turn pH‐ and temperature‐dependent, implying that delivery design must account explicitly for speciation at the exposure site.

Testing whether these principles translate to therapeutic benefit requires a defined experimental agenda. Priority directions include route‐specific pharmacokinetic studies quantifying systemic sulfide uptake and metabolism across immersion, inhalation, and oral hydropinotherapy, with standardized reporting of water temperature, measured sulfide concentration, and degassing conditions; randomized, double‐blind, controlled trials comparing sulfurous thermal water against heated non‐mineral water, using objective endpoints (e.g. blood oxidative‐stress biomarkers and validated functional measures such as lung‐function tests), to confirm any therapeutic efficacy attributable specifically to the sulfide content rather than to thermal or contextual effects; mechanistic studies—including in situ sulfide microelectrode profiling and ex vivo interface models—to test the bioglea's contribution to local sulfide dynamics and bioactivity; and adoption of a harmonized core outcome set and standardized exposure‐reporting framework for sulfurous balneotherapy, to enable cross‐study comparison and meta‐analysis. Pursued together, these directions would convert the descriptive observations summarized here into the quantitative, mechanism‐level evidence required to establish—or refute—a genuine translational role for sulfurous thermal systems.

## Author Contributions

**Elisabetta Ferrara:** conceptualization, investigation, writing – review and editing, writing – original draft. **Manela Scaramuzzino:** methodology, data curation, writing – original draft, writing – review and editing, validation. **Davide Ciaramellano:** writing – original draft, writing – review and editing. **Luigi Brunetti:** methodology, visualization, writing – review and editing, writing – original draft. **Giuseppe Balice:** writing – review and editing, writing – original draft. **Giovanna Murmura:** writing – review and editing, writing – original draft, visualization, data curation. **Bruna Sinjari:** data curation, supervision, writing – review and editing, writing – original draft.

## Funding

The authors have nothing to report.

## Conflicts of Interest

M.S. is affiliated with the Medical Thermal Center of Saturnia. Several authors have co‐authored prior observational studies conducted at the same facility (refs [26, 27, 28]), which are cited herein as hypothesis‐generating evidence; this potential interpretive bias is acknowledged in the main text. These relationships had no role in study design; in the collection, analysis, and interpretation of the literature reviewed; in the writing of the manuscript; or in the decision to submit it for publication. The remaining authors declare no conflict of interest.

## Transparency Statement

Elisabetta Ferrara affirms that this manuscript is an honest, accurate, and transparent account of the study being reported; that no important aspects of the study have been omitted; and that any discrepancies from the study as planned have been explained.

## Data Availability

Data sharing not applicable to this article as no datasets were generated or analysed during the current study.
